# Liver Graft-to-Spleen Volume Ratio as a Useful Predictive Factor of the Outcomes in Living Donor Liver Transplantation: A Retrospective Study

**DOI:** 10.3389/fsurg.2022.855695

**Published:** 2022-03-28

**Authors:** Fei Xiao, Lin Wei, Wei Qu, Zhi-Gui Zeng, Li-Ying Sun, Ying Liu, Hai-Ming Zhang, Yu-Le Tan, Jun Wang, Zhi-Jun Zhu

**Affiliations:** ^1^Liver Transplantation Center, National Clinical Research Center for Digestive Diseases, Beijing Friendship Hospital, Capital Medical University, Beijing, China; ^2^Department of Organ Transplantation, Liao Cheng People's Hospital, Liaocheng, China; ^3^Clinical Center for Pediatric Liver Transplantation, Capital Medical University, Beijing, China; ^4^Department of Critical Liver Diseases, Liver Research Center, Beijing Friendship Hospital, Capital Medical University, Beijing, China

**Keywords:** living donor liver transplantation, graft-to-spleen volume ratio, graft-to-recipient weight ratio, portal hypertension, splenectomy

## Abstract

**Background:**

In living donor liver transplantation (LDLT), graft-to-recipient weight ratio (GRWR) <0. 8% is an important index for predicted portal hypertension, which may induce the graft small-for-size syndrome (SFSS). Recently, the value of graft-to-spleen volume ratio (GSVR) on predicted portal hypertension had been reported, whether without splenectomy prevent portal hypertension in transplantation remains disputed, we aimed to identify GSVR contributing to portal venous pressure (PVP) and outcomes without simultaneous splenectomy in LDLT.

**Methods:**

A retrospective study had been designed. Excluded patients with splenectomy, 246 recipients with LDLT between 2016 and 2020 were categorized into a low GSVR group and a normal GSVR group. Preoperative, intraoperative, and postoperative data were collected, then we explored different GSVR values contributing to portal hypertension after reperfusion.

**Results:**

According to the first quartile of the distributed data, two groups were divided: low GSVR (<1.03 g/mL) and normal GSVR (>1.03 g/mL). For the donors, there were significant differences in donor age, graft type, liver size, GRWR, and GSVR (*P* < 0.05). Following the surgical factors, there were significant differences in blood loss and CRBC transfusion (*P* < 0.05). The low GSVR has demonstrated had a significant relationship with ascites drainage and portal venous flow after LDLT (*P* < 0.05). Meanwhile, low GSVR heralds worse results which covered platelet count, international normalized ratio (INR), and portal venous velocity. Kaplan–Meier analysis showed that there was a significant difference between the two groups, while the low GSVR group demonstrated worse recipients survival compared with the normal GSVR group (*P* < 0.05).

**Conclusions:**

Without splenectomy, low GSVR was an important predictor of portal hypertension and impaired graft function after LDLT.

## Introduction

Liver transplantation is an effective treatment for end-stage liver disease. However, due to the shortage of organs in China, living donor liver transplantation (LDLT) has become the best choice for end-stage liver disease patients. Small-for-size syndrome (SFSS) is a complication that may induce severe outcomes after LDLT (1). It is well known that the characteristics of SFSS are hyperbilirubinemia (>5.8 mg/dL), international normalized ratio (>2), encephalopathy (grade III/IV), and ascites drainage (>1,000 mL) (2). Portal hypertension plays a key factor in SFSS, and it may persist as massive ascites, hyperbilirubinemia, and coagulopathy, also associated with a lower recipient survival (3). Portal venous pressure consists of 3 elements: intrahepatic vascular resistance, outflow, and hemodynamic status. Intrahepatic vascular resistance is related to the volume and quality of the graft. Outflow is affected by the construction of the hepatic vein. Hemodynamic status is affected by the spleen volume and intestinal membrane blood vessels (4, 5). During Pre-LDLT evaluation, graft-to-recipient weight ratio (GRWR) is an important index predicted portal hypertension (6, 7). Traditionally, GRWR > 0.8% in adult live liver transplantation may prevent the SFSS, while live liver transplantation in children needs to be maintained at 2–4%. But portal hyperperfusion was still found in recipients even though the satisfied GRWR presented during LDLT. Portal venous flow (PVF) and ascites are convenient to record during post-LDLT. In our reviews, portal hyperperfusion may occur even if an adequate graft size was applied, which is defined as a PVF ≥250 ml/min/100 g graft. Previous research has shown that the spleen volume was significantly associated with an excessive PVF, then, the value of graft-to-spleen volume ratio (GSVR) on predicted portal hypertension had been reported (8).

Considering previous studies (9, 10), it seems that GSVR heralds portal hypertension after LDLT, but varieties of liver diseases and surgical schemes mean different cut-off values about the GSVR, whether without splenectomy prevent portal hypertension in transplantation remains disputed. Our study was conducted to evaluate the clinical impact of low GSVR after LDLT without splenectomy.

## Materials and Methods

A single-center retrospective analysis was performed, including all patients who underwent LDLT at the Beijing Friendship Hospital, China, between January 2016 and April 2020. The study was approved by the Beijing Friendship Hospital Research Ethics Committee (Approval Number: 2021-P2-409-01). A total of 238 patients were enrolled after excluding the following cases: 5 with splenectomy during operation, 25 without whole spleen imaging by preoperative computed tomography, and 80 with incomplete clinical data.

Preoperative imaging evaluation of the patients was performed using multislice spiral CT (Siemens, Germany). Compared to the actual liver weight measured during the operation, the evaluation of the influence of the donor's liver volume before the operation is more valuable. The liver and spleen volumes were measured by hand tracing the portal venous phase images in the CT examination with the major vessels were excluded. The area of the organ in each section was multiplied by the slice thickness to calculate the volume. The total volume of the organ was then calculated by adding the individual volumes. The liver and the spleen have similar density as water, so graft weight was defined as the graft volume (11). The GSVR and graft-to-recipient weight ratio (GRWR) follow the volume based on imaging. Portal flow velocity was measured after arterial reconstruction by Doppler. The portal vein flow (PVF) was measured by recording the area of the portal vein and recorded as ml/min/100 g graft.

Primary non-function (PNF) is defined as a clinical disorder that results in liver necrosis or multisystemic dysfunction which usually requires liver retransplantation (during the 90 days) (12). Unplanned reoperation was defined as forced to surgery again because of complications after transplantation. The decision regarding a right or left liver graft and whether to include the middle hepatic vein (MHV) or not was taken before surgery.

All recipients underwent the same surgical procedure. Portal vein anastomosis was done using 6-0 or 5–0 Prolene sutures in a running fashion with a growth factor. Hepatic artery anastomosis was done using interrupted 8-0 or 7–0 Prolene sutures. Biliary drainage was established by duct-to-duct or by duct-to-Roux-en-Y drainage. The drainage tube was kept unobstructed after the operation and the ascites volume was recorded daily. The immunosuppressive treatment protocols included methylprednisolone and tacrolimus. The first methylprednisolone dose (10 mg/kg) was preferred after the surgery. Dose tapered from 10 to 0.1 mg/kg/day during the postoperative period, using <3 months in duration. Tacrolimus (0.01–0.05 mg/kg/day) was the first choice for immunosuppressive therapy at the third-day post-LDLT and the mycophenolate mofetil (10−30 mg/kg/day) were initiated on postoperative day 3. The methylprednisolone trough level of tacrolimus was adjusted to 8–10 ng/ml (13, 14).

Focusing on the prognosis of liver transplantation, we intendedto describe a cut-off value of GSVR; patients were separated into two groups (normal GSVR group and low GSVR group).

### Statistical Analyses

Continuous data are expressed as medians with ranges or interquartile ranges (IQRs) as appropriate. Categorical data are presented as numbers and percentages. Comparisons were expressed by the Mann–Whitney *U*-test for continuous variables and the Chi-square test for categorical variables as appropriate. The PNF was analyzed using the univariate analysis, and variables significant at a *p* < 0.20 in the univariate analysis were used in the multivariate logistic regression model (15). Graft survival was estimated by the Kaplan–Meier method and differences in survival between the two groups were compared with the log-rank test. A value of *p* < 0.05 was considered to indicate statistical significance. Pearson correlation coefficient was used to determine the relationship between drainage, portal flow, and GSVR.SPSS21.0 (IBM, United States) was used for all statistical analyses.

## Results

**Figure 1:**
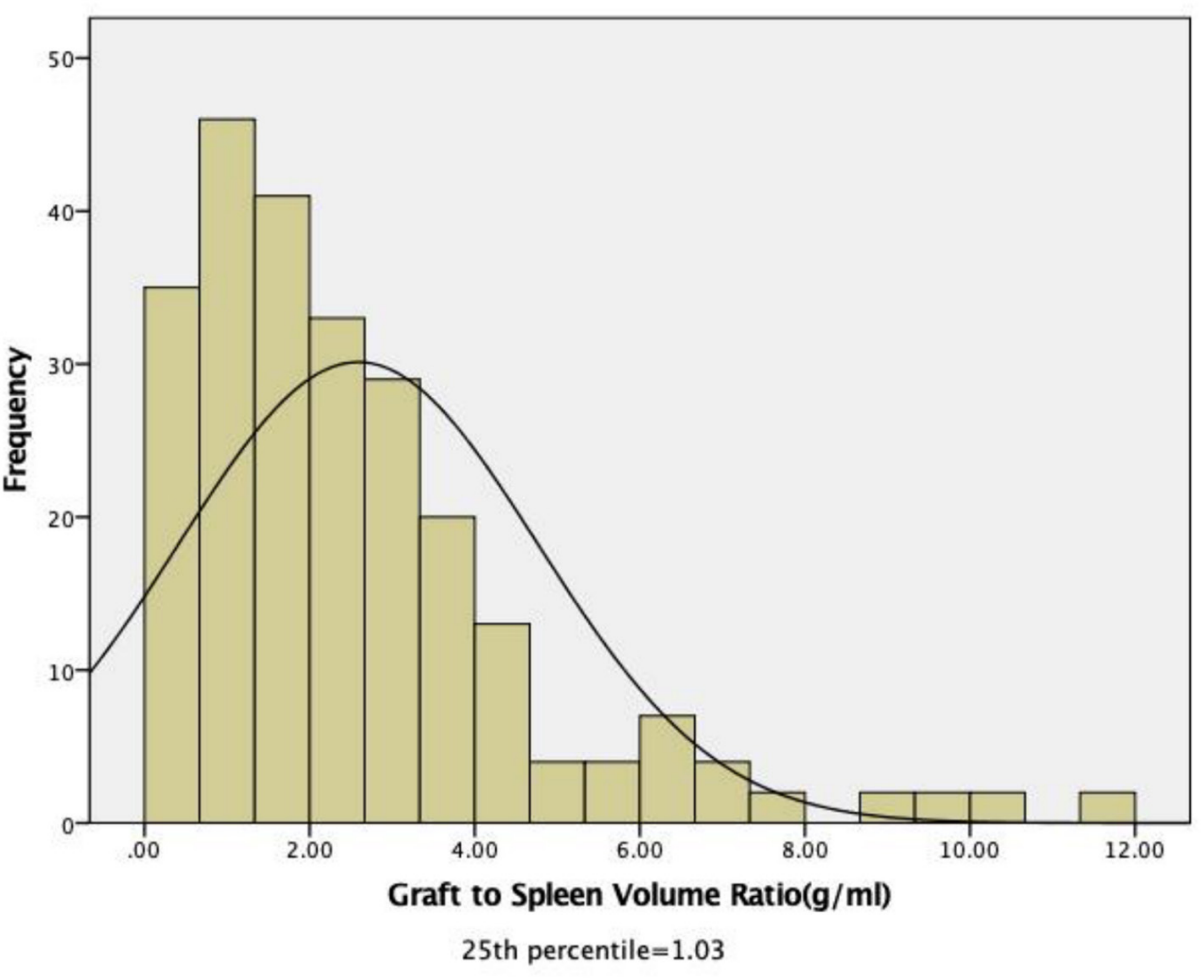
Distribution of the pretransplant graft-to-spleen volume ratio among all recipients. The median GSVR in the whole cohort was 2.02 g/mL (range: 0.23–11.99). The 1st and 3rd quartiles were 1.03 and 3.37 g/mL.

The median GSVR in the whole cohort was 2.02 g/ml (range, 0.23–11.99). The 1st and 3rd quartiles were 1.03 and 3.37 g/ml. The cut-off value according to the IQR is based on 246 measured values. According to the research of previous scholars, low GSVR lead to bad endings; then the 1st quartile as the threshold, recipients were assigned to two groups: low GSVR (<1.03 g/ml, *n* = 59) or normal GSVR (>1.03 g/ml, *n* = 187) (Figure 1).

### Characteristics of Patients and Outcomes

All recipients (children and adults) are described in Table 1. A total of 246 patients were included in the study. There were 125 male (50.8%) and 121 female (49.2%) recipients. The diagnoses of patients were as follows: biliary atresia (73.2%), other cholestatic liver diseases (8.9%), metabolic diseases (5.7%), acute liver failure (2.0%), and others (10.2%). We discriminated the graft types of donors as follows: left lateral (73.2%), left lobe with MHV (8.9%), left lobe without MHV (5.7%), right lobe with MHV (2.1%), right lobe without MHV (7.3%), domino liver (2%), segment II (0.8%). There were significant differences in recipient age, BMI, variations in liver disease, and spleen size (*P* < 0.05). The median age and spleen size were significantly larger in the normal GSVR group than in the low GSVR group. For the donors, there were significant differences in donor age, graft type, liver size, GRWR, and GSVR (*P* < 0.05). Regarding the surgical factors, there were significant differences in blood loss and CRBC transfusion (*P* < 0.05). Thus, there were no differences in postoperative outcomes.

**Table 1:** Clinical characteristics of recipients, donors, surgical factors and outcomes according to graft-to-spleen volume ratio.

**Variables**	**Total *n* = 246**		
	**GSVR>1.03**	**GSVR <1.03**	***P***
	***n* = 187**	***n* = 59**	**value**
**Recipients**			
Age, month	13 (7–42)	92 (50–146)	0.000
Sex (*n*, %)			0.230
Male	91 (48.7)	34 (57.6)	
Female	96 (51.3)	25 (42.4)	
BMI	16.2 (14.8–17.8)	16.9 (15.4–19.5)	0.047
Diagnose (*n*, %)			0.000
Biliary atresia	154 (82.4)	26 (44.1)	
Cholestatic liver diseases	8 (4.3)	14 (23.7)	
Metabolic diseases	10 (5.3)	4 (6.8)	
Acute liver failure	0 (0)	5 (8.5)	
Others	15 (8.0)	10 (17)	
Child-Pugh score	9 (7–11)	9 (8–10)	0.685
PELD score	16 (10–22)	17 (10–23)	0.700
MELD score	18 (14–22)	19 (15–23)	0.438
Spleen size, cm3	115.7 (76.5–192.5)	594.3 (432.7–1,136.0)	0.000
**Donors**			
Age,month	382 (345–436)	408 (359–457)	0.005
Sex (*n*, %)			0.399
Male	91 (48.7)	25 (42.4)	
Female	96 (51.3)	34 (57.6)	
Graft type (*n*, %)			0.000
Left lateral	154 (82.4)	26 (44.1)	
Left lobe (with MHV)	8 (4.3)	14 (23.7)	
Left lobe (without MHV)	10 (5.3)	4 (6.8)	
Right lobe (with MHV)	0 (0)	5 (8.5)	
Right lobe (without MHV)	9 (4.8)	9 (15.3)	
Domino liver	4 (2.1)	1 (1.7)	
Segment II	2 (1.1)	0 (0)	
liver size,cm3	293.5 (260.1–345.1)	354.4 (289.1–476.4)	0.015
GSVR,g/ml	2.62 (1.79–3.79)	0.60 (0.40–0.83)	0.000
GRWR,%	3.09 (2.22–4.12)	1.56 (1.18–2.15)	0.000
**Surgical factors**			
ABO incompatibility (n, %)	20 (10.7)	3 (5.1)	0.093
CIT,min	88 (76–107)	89 (67–107)	0.906
WIT,min	2 (0–3)	3 (0–3)	0.747
Blood lose,ml	150 (100–300)	300 (150–700)	0.000
CRBC transfusion,U	1 (0–2)	2 (0–4)	0.008
**Outcomes**			
Postoperative hospital stay,d	21 (16–28)	23 (14–36)	0.220
Drainage tube stay,d	12 (9–16)	13 (8–18)	0.261
Drainage tube stay>14,d	59 (31.6)	20 (33.9)	0.736
Drainage tube stay>30,d	15 (8.0)	7 (11.9)	0.367
Complications (*n*, %)			
Hemorrhage			0.890
without operation	2 (1.1)	1 (1.7)	
operation	5 (2.7)	2 (3.4)	
Early graft loss			0.455
Infection	11 (5.9)	6 (10.2)	
Bleeding	1 (0.5)	0 (0)	
Unplanned reoperation (*n*, %)			0.126
Bile leakage	1 (0.5)	2 (3.4)	
Bile duct stricture	4 (2.1)	4 (6.8)	
Bleeding	2 (1.1)	2 (3.4)	
Bowel perforation	7 (3.7)	2 (3.4)	
PVT	2 (1.1)	2 (3.4)	
PVS	7 (3.7)	0 (0)	
HAT	3 (1.6)	0 (0)	
Intestinal obstruction	1 (0.5)	0 (0)	
Mortality (*n*, %)	13 (7.0%)	6 (10.2%)	0.420

*GRWR, Graft-to-recipient weight ratio; GSVR, graft-to-spleen volume ratio; CRBC, Concentrated red blood cells; WIT, Warm ischemia time; CIT, Cold ischemia time; HAT, Hepatic artery thrombosis; PVT, Portal vein thrombosis; PVS, Portal vein stenosis*.

**Figure 2:**
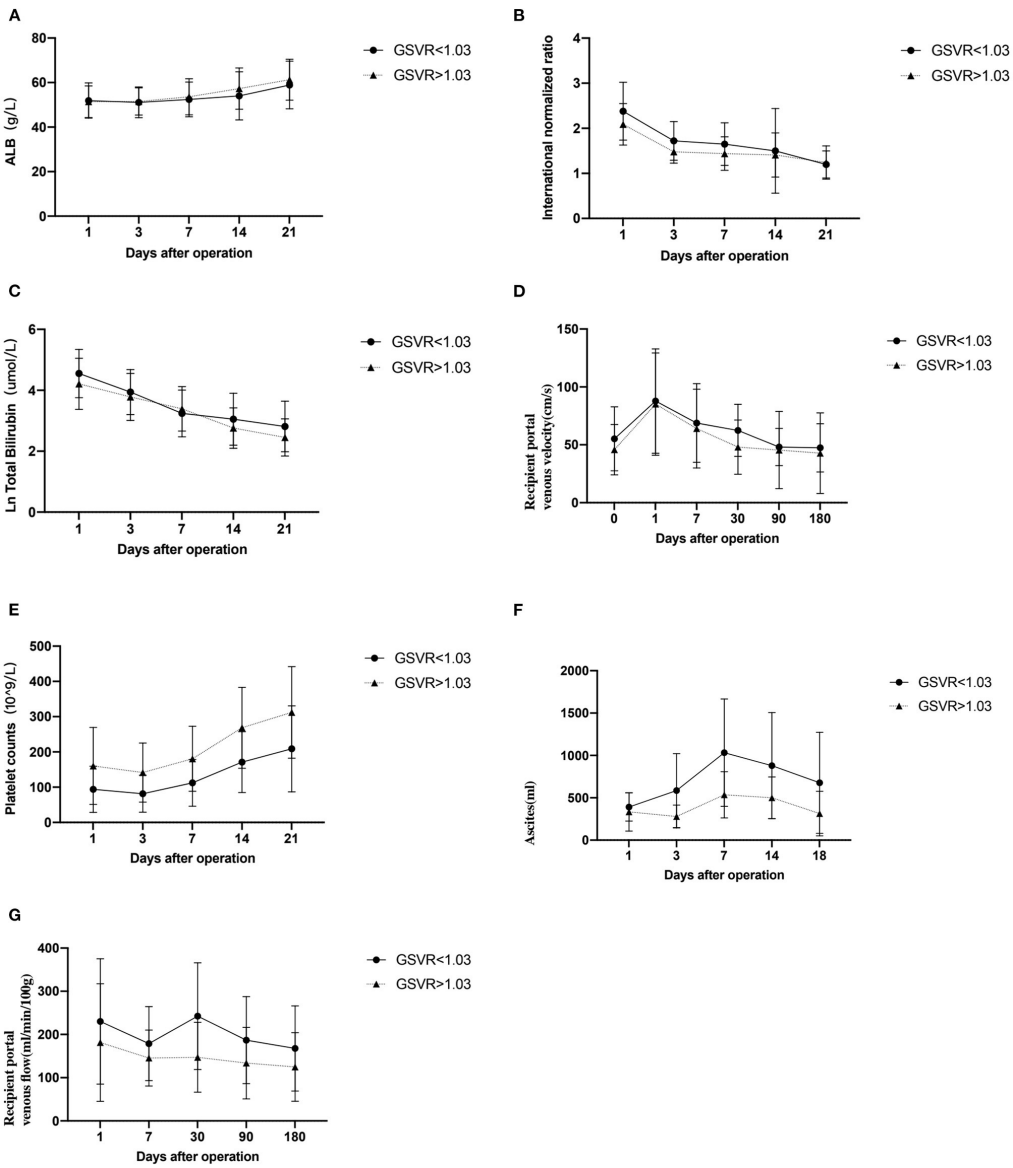
The clinic data according to graft-to-spleen volume ratio **(A–G)**.

Figures 2A–G summarizes the postoperative clinic data according to the GSVR. We recorded the total bilirubin (TB), platelet count, international normalized ratio (INR), albumin (ALB), and ascites in the first month after LDLT. Recipients were attached PVF (ml/min/100 g) and portal venous velocity (PVV cm/s) in the 6 months after LDLT. There were no significant differences in the ALB, INR, TB, and PVV between the normal GSVR and low GSVR groups (Figures 2A–D). The ALB remained increased on Day 1 after the surgery in the two groups, whereas the INR and TB diminished afterward. PVV rapidly increased during post-LT Day 1, thus diminished in the following 6 months. There were significant differences in the platelet count, ascites, and PVF between the normal GSVR and low GSVR groups (Figures 2E–G). The ascites in both the groups gradually increased until post-LT week 1, and then reduced until the drainpipe was removed. Regarding the PLT, it remained as a low median value and required more than 1 week until it normalized in the low GSVR group. Similarly, PVF in the low GVSR group was higher than the normal group and maybe caused by higher flow in the portal vein system.

### Relationship Between PVF, Drainage, and GSVR

**Figure 3:**
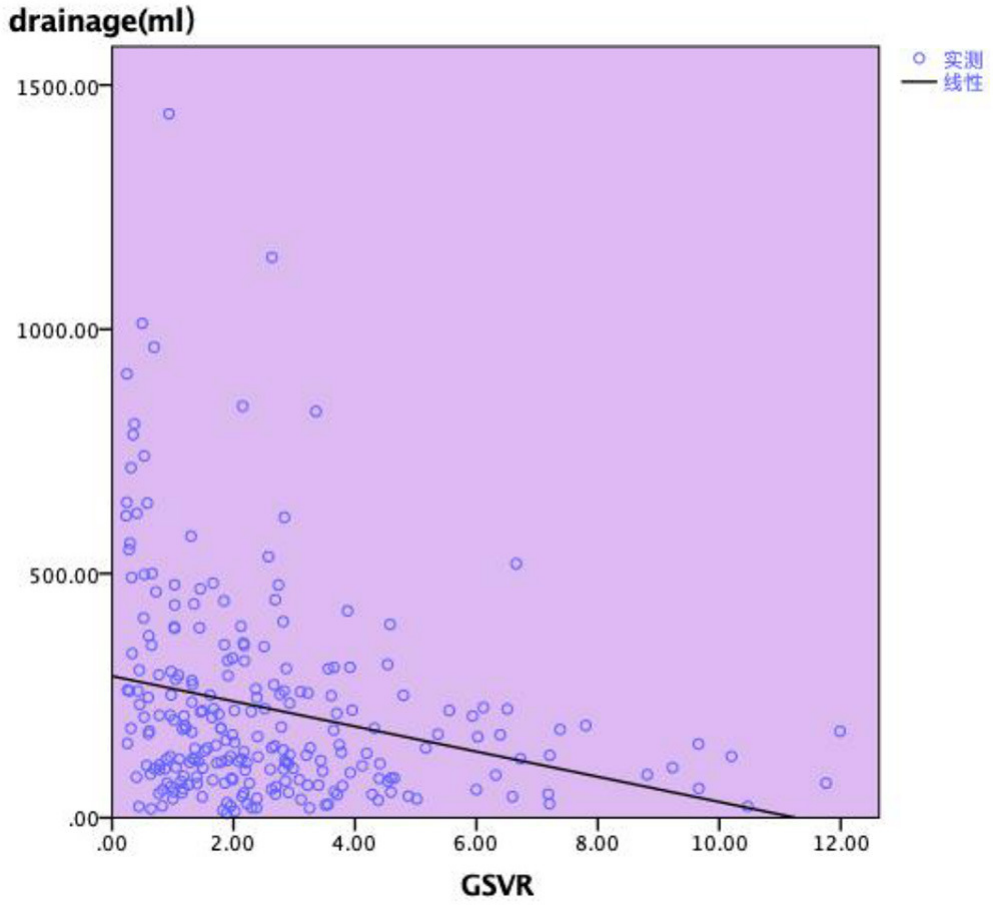
Relationship between GSVR and drainage after transplantation.

**Figure 4:**
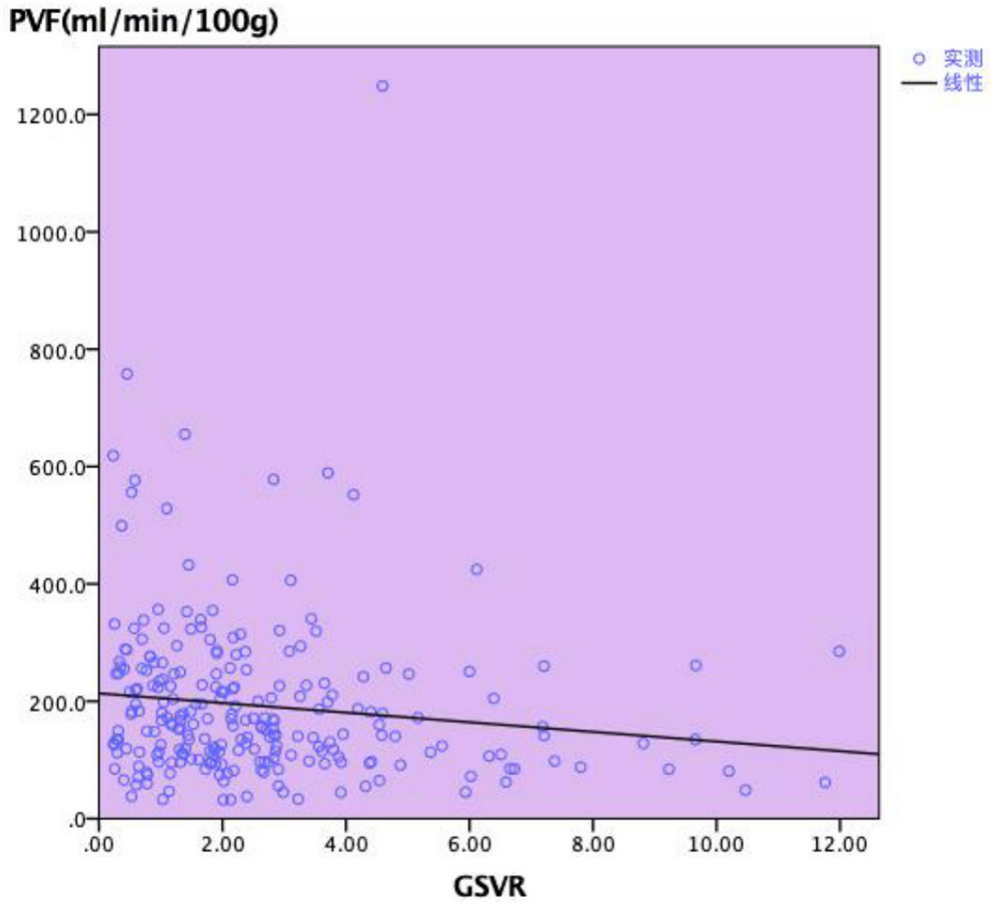
Relationship between GSVR and PVF after transplantation.

Ascites volume was recorded daily after surgery, and the average of the volume (drainage) was calculated. As shown in Figure 3, there was a statistically significant relationship noted between GSVR and drainage (*p* = 0.001). This suggested that GSVR might be a useful predictor of portal hyperperfusion syndrome in LDLT, while for the recipients, low GSVR presumes to extend the drainage tube time. Portal flow velocity was measured after arterial reconstruction by Doppler the first day after LDLT, all the recipients had been attached PVF. The GSVR showed a positive correlation with the PVF after LDLT. There was a statistically significant relationship noted between GSVR and PVF (*p* = 0.043; Figure 4).

### Risk Factors for PNF and Survival Analysis

Risk factors for PNF were assessed among the two groups. The graft-to-recipient weight ratio (GRWR) was analyzed with Cox proportional hazards models (Table 2). Multivariable logistic regression model analysis revealed that GRWR (HR, 2.785; 95%CI, 1.134–6.893; *p* = 0.025) independently affected early graft survival after liver transplantation.

**Table 2:** Baseline risk factors for PNF that were included in the multivariate logistic regression model.

**Variables**	**Univariable HR (95%CI)**	***P* value**	**Multivariable HR (95%CI)**	***P* value**
Age	0.996 (0.988–1.004)	0.354		
BMI	1.020 (0.850–1.224)	0.832		
Child-Pugh C	1.520 (0.851–2.713)	0.157	1.632 (0.913–2.824)	0.100
MELD > 25	0.924 (0.810–1.054)	0.239		
Spleen size,by 100 ml	1.003 (1.000–1.006)	0.074	1.012 (0.973–1.022)	0.068
GSVR	0.656 (0.298–1.446)	0.296		
GRWR	2.526 (2.652–7.132)	0.047	2.785 (1.134–6.893)	0.025
CIT > 60 min	1.008 (0.996–1.020)	0.191	1.114 (0.942–1.189)	0.131

*MELD, Model for End-stage Liver Disease; GRWR, Graft-to-recipient weight ratio; CIT, Cold ischemia time; GSVR, Graft-to-spleen volume ratio; HR, Hazard ratio; CI, Confidence interval; PNF, Primary non-function*.

**Figure 5:**
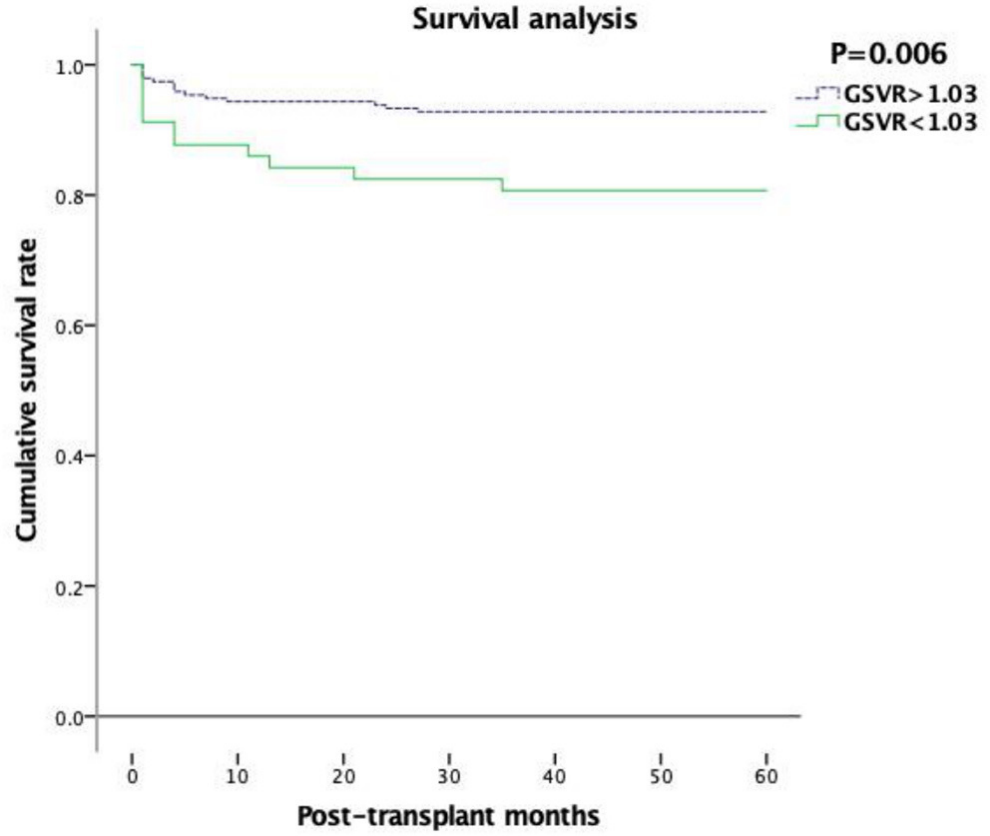
Graft survival according to graft-to-spleen volume ratio.

Kaplan–Meier analysis shows that there was a significant difference between the two groups as the low GSVR group demonstrated worse recipients survival compared with the normal GSVR group (*p* = 0.006) (Figure 5).

## Discussion

Liver transplantation is an effective treatment for end-stage liver disease. However, due to the shortage of organs in China, LDLT has become the prior choice for patients with end-stage liver disease. This study included a variety of diseases, including biliary atresia and liver failure, represented by cirrhosis, and metabolic diseases, represented by non-cirrhosis. Hypersplenism function and volume increase are common complications in patients with liver cirrhosis. At the same time, the blood flow of the spleen contributes 52% of the blood flow of the portal vein of the liver (16). Therefore, the size of the spleen can reflect the severity of portal hypertension before transplantation. Although the GV/SLV and GRWR are generally accepted as important predictors of the adequacy of the post-transplant liver function (17), we have encountered some patients whose post-transplant recovery was not good despite meeting these criteria. GSVR has been affirmed as a new marker to predict liver function recovery indicator after LDLT, but different research centers have given various research results, and the types of diseases included are not consistent. Therefore, we designed a project that includes multiple diseases cohort study to observe the prognostic effect of GSVR indicator in LDLT.

First, we focused on the clinical recovery after LDLT. We defined the GSVR as 1.03, the patients were divided into 2 groups (GSVR > 1.03 and GSVR <1.03), and the observation of total protein, bilirubin, ascites, INR, and PLT have been recorded. These indicators represent the recovery of liver function and the possibility of small liver syndrome. Yao (10) deemed low GSVR was associated with thrombocytopenia, hyperbilirubinemia, coagulopathy, and massive ascites, which lead to a poor prognosis, Small-size liver leads to the insufficient functional liver volume of the graft. The main physiological basis for its occurrence is excessive portal vein perfusion and continuous portal hypertension. When the portal vein blood flow increases too much, it will directly cause mechanical damage to hepatic sinusoidal endothelial cells and portal vein endothelial cells, and cause vasoconstriction and relaxation disorders, leading to sinusoidal microcirculation disorders (18). In addition, excessive portal blood flow will reduce the hepatic arterial blood flow through the buffering effect of the hepatic artery (19), which will cause a series of ischemic damage, sheet necrosis of liver tissue, and bile duct cell ischemia. Eventually lead to a series of clinical manifestations, such as increased blood bilirubin, prolonged clotting time, and massive ascites. Increased portal pressure can lead to hypersplenism function and further reduce the number of platelets in the circulation, increasing the risk of bleeding. Lesurtel (20) pointed out that thrombocytopenia may prevent liver regeneration, which leads to the potential for SFSS. In combination with our research, platelets tended to decline 3 days after surgery, which may be related to platelet consumption caused by bleeding from the wound after surgery. Three days later, the platelet count gradually increased; however, the platelet count of the low GSVR group continued to be lower than that of the normal GSVR group. There was no significant difference between the two groups after surgery. Enhanced recovery after surgery protocols in patients undergoing liver transplantation: the ALB is supplemented with intravenous fluids and intestinal nutrition to maintain a high level. Increasing the level of ALB after surgery is beneficial to reduce the number of ascites and restore liver function. Our research suggests that the volume of ascites in the low GSVR group is significantly higher than that in the normal group, and two groups have a common trend: the volume of ascites reaches the highest value within 7 days after surgery, and then gradually decreases. Regarding TB and INR, there was no significant difference between the two groups. After the operation, they showed a gradual decline with the recovery of liver function. To prevent vascular thrombosis, postoperative anticoagulation therapy affected the INR but did not affect its recovery trend. Although the difference was not significant, the low GSVR group showed better recovery of liver function and lower INR than the normal group.

Studies have revealed that high PVP after reperfusion is a key factor in the occurrence of SFSS. As we know, three factors reflect PVP: the size of the graft, outflow from the hepatic vein, and portal hemodynamic status. Since PVP was not tested during and after the operation, in this study we focused on the volume of ascites, the average catheterization time, and PVF of the patients after the operation. Vasavada (21) believed that a PVF immediately after reperfusion > 190/ml/min/100 g predicted graft SFSS, yet, Kato et al. (22) showed a portal flow greater than 250 ml/min/100 g may lead to SFSS. Gyoten (9) believed that GSVR <0.95 predicts portal hypertension of more than 20 mmHg in adult-to-adult LDLT, Cheng (8) deemed a GSVR <0.6 was highly associated with posttransplant elevated PVF. If the GSVR was less than 0.6, there is a high possibility of excessive portal flow; this linear relationship also explained our research results. Meanwhile, our study observed the post-LT PVV (cm/s) – low GSVR group showed the faster blood flow velocity than the normal GSVR group, although the gap between the two groups is not obvious. GSVR may be a useful indicator that induces the recipient's portal hyperperfusion syndrome which leads to a SFSS. The number of researchers included in the study is small, which shows the different cut-off values of GSVR compared to our results. Similarly, Figure 3 demonstrates there is a statistically significant relationship between GSVR and drainage. The low GSVR group tends to have more ascites volume and tube days after surgery. Meanwhile, the low GSVR group showed a higher ratio than the normal group for drainage tube stay >14 days and drainage tube stay > 30 days, indicating that low GSVR may lead to high PVP after LDLT, which may further induce SFSS.

This study did not detect intraoperative portal pressure, as the replacement, Doppler has been used to check the postoperative liver portal vein blood flow and flow rate, which is an indirect reflection of the portal vein pressure, but this examination reduced the accuracy of the portal vein pressure, and brought difficulty in the judgment of postoperative ascites volume and SFSS. In this study, we excluded patients who underwent splenectomy due to hypersplenism. As our study showed, it took a long time for the platelets to return to normal and longer drainage tube days in the low GSVR group. During the operation, we faced the challenge of deciding whether to perform splenectomy or splenic artery modulation during the operation. Umeda (23) reported that preoperative embolization of the splenic artery and intraoperative ligation of the splenic artery taken the same effect on the adjustment of portal blood flow, and pointed out that it will not harm the graft regeneration like the inferior vena cava portal vein shunt does. Humar (24) believed that when SFSS has occurred after surgery, therapeutic splenic artery ligation or embolization of the splenic artery through intervention can also be effective in treating the small liver syndrome. Therefore, splenic artery ligation or embolization becomes an important therapy for small liver syndrome (25). However, the adjustment of splenic artery ligation on portal hyperperfusion is limited, and its reduction in PVP is usually about 2 mmHg. Splenectomy can effectively reduce portal vein blood flow and portal vein pressure, thereby reducing the possibility of SFSS after LDLT. Thus, previous studies (26) indicated that liver transplantation meanwhile splenectomy may be due to pancreatic injury, infection, and thrombosis, and the use of postoperative immunosuppressants increases this uncertainty. Yoshizumi (27) deemed that simultaneous splenectomy improves outcomes after adult LDLT. Wei (28) reported that partial splenectomy was performed in children's LDLT to cope with hypersplenism. This surgical method effectively reduced the hypersplenism and preserved the physiological function of the spleen, and achieved active results after the operation. Therefore, we may apply partial splenectomy or splenic artery ligation to reduce portal blood flow. We shall design a corresponding study to observe the effect of simultaneous removal of the spleen or without removal of the spleen during the operation on different GSVR groups for further research.

The follow-up results of our study suggested that the low GSVR group showed a worse prognosis, and it showed more obviously within 12 months after LDLT. Interestingly, according to multivariate analysis, GRWR is an important factor of PNF (*P* < 0.05), and GSVR <1.03 g/ml is not a key factor of PNF. Previous results (29) suggest that the incidence of PNF is between 15 and 27% after LDLT. According to Abu-Gazala's (30) definition, some recipient factors (lower age and high scores of BMI, Child–Pugh, and MELD), donor factors (younger age), and transplant factors (prolonged ischemic time) are relevant to PNF formation. Our study demonstrated a lower incidence (6.9%) after LDLT, and mainly due to postoperative infections, which may be due to those before us for liver surgery having developed the strict standard, skilled surgical techniques to shorten the warm and cold ischemia and ischemia time and elaborated postoperative management. Our study demonstrated a lower PNF incidence (6.9%) after LDLT, and mainly due to postoperative infections, which may be due to those before us for liver surgery having developed the strict standard, skilled surgical techniques to shorten the warm and cold ischemia and ischemia time and elaborated postoperative management.

Our research focuses on patients with LDLT. Although domino liver transplantation was included, the number was small. Therefore, it is necessary to design a cohort study of partial liver transplantation and whole liver transplantation to explore the differences regarding GSVR in different liver transplant types. In addition, this study included patients with cirrhosis of the liver with mainly biliary atresia and patients with non-cirrhosis of the liver with metabolic diseases. Therefore, whether there are differences in GSVR in different disease types also needs further research.

## Conclusions

This study explored the prognosis of GSVR in LDLT. However, it has not been proved that it directly causes small liver syndrome and PNF. Nevertheless, GSVR was an important predictor of portal hypertension and impaired graft function after LDLT. Its significance lies in judging GSVR through imaging before the operation, eliminating the need for intraoperative portal vein pressure measurement. In addition to GRWR, an evaluation parameter that has a good reference value for prognosis has been added. Although we do not agree with the resection of the spleen, it provides a reference for judging the portal vein blood flow, handling the branches of the portal vein, and whether to perform partial splenectomy during the LDLT.

## Data Availability Statement

The original contributions presented in the study are included in the article/supplementary material, further inquiries can be directed to the corresponding author.

## Ethics Statement

The studies involving human participants were reviewed and approved by Beijing Friendship Hospital Research Ethics Committee. Written informed consent from the participants' legal guardian/next of kin was not required to participate in this study in accordance with the national legislation and the institutional requirements.

## Author Contributions

FX, Z-JZ, LW, and WQ contributed to conception, design, and surgeries. H-MZ, Y-LT, and Z-GZ contributed to collection and assembly of data. L-YS, YL, and JW contributed to data analysis and interpretation. All authors contributed to provision of study materials or patients and manuscript writing.

## Funding

This study was supported by grants from the Capital's Funds for Health Improvement and Research (No. 2020-1-2024).

## Conflict of Interest

The authors declare that the research was conducted in the absence of any commercial or financial relationships that could be construed as a potential conflict of interest.

## Publisher's Note

All claims expressed in this article are solely those of the authors and do not necessarily represent those of their affiliated organizations, or those of the publisher, the editors and the reviewers. Any product that may be evaluated in this article, or claim that may be made by its manufacturer, is not guaranteed or endorsed by the publisher.
